# Kuemmell’s Mercuric Bi-Chloride Antiseptic Method*Archiv fur klinische Chirurgie. Band xxviii., Heft 3, pp, 673–719.

**Published:** 1883-10

**Authors:** 


KUEMMELL'S MERCURIC BI CHLORIDE ANTISEPTIC METHOD.*—Corrosive sublimate, or, according to the more modern nomenclature, mercuric bi-chloride, is among the oldest antiseptic preparations whose superior anti-putrefactive properties were well known. But there seems to have been no disposition among surgeons to put it to practical use in the treatment of wounds, probably through fear of its well known toxic effects. Nor need one wonder at this when the maximum dose of the drug is taken into consideration, and the certainty with which alarming symptoms of poisoning are produced when this dose is exceeded. The first clinic in which the use of it was ventured in any shape was that of Von Bergman, of Würtzberg, where a gauze prepared with it was used instead of a carbolized gauze.

The credit of using it as a general antiseptic, both for purposes of irrigation and wound dressing, belongs to Kümmell, of Hamburgh, who, having his attention called to its powerful germicide properties by the labors of Koch, proceeded by a series of experiments to demonstrate in how far it might be made useful to the operating surgeon. Dougall, Billroth, Buchholtz and Sternberg, of the United States Army, found that in solutions varying in strength from 1 to 1.000 to 1 to 20.000 bacteria were killed and their further development checked. The bacillus of gangrene of the spleen, according to Koch and Pasteur, resists the action of all other antiseptics with the exception of that of the mercuric bichloride. In a solution of this latter of the strength of 1 to 1,000 Koch succeeded in destroying them entirely within a few minutes. In a solution of the strength of 1 to 5,000 their growth was markedly retarded.

Acting upon the possibilities which these experiments suggested, Kümmell proceeded to make a practical test of the applicability of the mercuric bichloride as an antiseptic wound dressing. Disappointed at the comparatively slight antiseptic effect of the five per cent. carbolic solution in general use for purposes of irrigation, he at first used a 1 to 5,000 solution of mercuric bichloride for the same purpose in Schede's wards in the Hamburgh General Hospital. He gradually increased the strength of

*Archiv fur klinische Chirurgie. Band xxviii., Heft 3, pp, 673–719.

the irrigating fluid to 1 to 1,000, and even to a one per cent. solution, without the slightest trace of dangerous symptoms supervening. The five per cent. solution of carbolic acid was used for the spray and as a bath for the instruments. The latter, if exposed to the action of the mercuric bichloride for only a few seconds, will become blackened by the rapid amalgamation upon their bright surfaces; nickle plating will not protect them from this injury. Enveloped as the head of the operator and his assistants are by the spray, a sufficient amount of the latter, were it to be even an atomization of a very dilute solution of the mercuric bichloride, might be productive of troublesome if not positively dangerous symptoms. It is evident, therefore, that for neither of the above purposes can the corrosive sublimate solutions be made available. In two patients treated with the one per cent. solution the constitutional effects of the drug appeared; in one salivation taking place, and in the other a diarrhœa, which latter was afterwards thought to be of a tuberculous origin. Recovery took place in both cases in a few days without the necessity of a removal of the dressings. Both of these patients had suffered previously from iodoform intoxication, suggesting a peculiar susceptibility to the action of antiseptics.

Sine the introduction of the mercuric bichloride solution as an irrigating fluid, Kümmell deprecates the use of sponges, except when absolutely required in the operation itself; the cheapness of the sublimate justifies its free use in cleansing the parts. The floor and the walls of the operating room are scoured and scrubbed with the solution, and no accident has yet occurred to either attendants or patients. After seven months use of the substance in this free manner, he avers that, with the exception of the two cases above alluded to, no ill effects whatever have been attributed to it.

In a person with an extremely sensitive skin the dressings of mercuric bichloride may, according to Kümmell, produce an eczematous condition of the parts surrounding the wound. This does not occur from a simple irrigation of the wound and the surrounding tissues; but when it does take place, which is rare, it arises from the constant contact of the wound and its neighborhood with dressings impregnated with the sublimate. In wounds in which putrefaction changes have already occurred, the mercuric bichloride solution quickly banishes the odor and arrests the septic processes.

The dresssings devised by Kümmell consist of sublimated gauze and cotton, sublimated silk, sublimated cat-gut, sublimated oil, and sublimated inorganic dressing materials. These latter comprise powdered glass, sand, coal ashes, asbestos, lint made from spun glass, and, for purposes of drainage, capillary threads of spun glass.

Sublimated gauze and cotton are designed to take the place of carbolized gauze and cotton. They are made hygroscopic in the usual manner, and then impregnated with the following:

| | |
|---|---|
| Corrosive sublimate...................... | 10 parts. |
| Alcohol....................................... | 4,490 " |
| Glycerine .................................. | 500 " |

The moisture is pressed out with a clothes wringer. If a stronger solution of the corrosive sublimate is used, eczema and bullæ of the integument will be produced.

For sutures, sublimated silk is used. It is previously prepared as for carbolized silk, by Hegar's method, and then boiled for two hours in a one per cent. solution of the corrosive sublimate; it is then transferred to a one-tenth of one per cent. solution of the same, where it is kept ready for use.

For ligatures sublimated cat-gut is used. The unprepared cat-gut is first immersed in a one per cent. solution of the corrosive sublimate for twelve hours, and then firmly wound upon spools, and preserved in an alcoholic solution of half of the above strength, to which is added ten per cent. of glycerine. Cat-gut thus prepared is very flexible, and it is asserted by Kümmell will give perfect immunity against infection, whatever might have been the condition of the gut prior to its preparation.

Sublimated oil of the strength of one per cent. is employed for uniformity's sake. This oil, however, as shown by Wolfhügel and Knorre, of the Imperial German department of health, possesses no more powerful antiseptic properties than carbolized oil. —*Annals of Anatomy and Surgery.*

